# Polyneuropathy in systemic sclerosis: exploring the causes and biomarkers

**DOI:** 10.3389/fmed.2024.1412706

**Published:** 2024-08-02

**Authors:** Kristīne Ivanova, Maksims Zolovs, Kaj Blennow, Henrik Zetterberg, Nataļja Kurjāne, Viktorija Ķēniņa

**Affiliations:** ^1^Department of Doctoral Studies, Rīga Stradinš University, Rīga, Latvia; ^2^Department of Rheumatology, Pauls Stradiņš Clinical University Hospital, Rīga, Latvia; ^3^Statistics Unit, Rīga Stradinš University, Rīga, Latvia; ^4^Institute of Life Sciences and Technology, Daugavpils University, Daugavpils, Latvia; ^5^Department of Psychiatry and Neurochemistry, Institute of Neuroscience and Physiology, the Sahlgrenska Academy at the University of Gothenburg, Mölndal, Sweden; ^6^Clinical Neurochemistry Laboratory, Sahlgrenska University Hospital, Mölndal, Sweden; ^7^Paris Brain Institute, ICM, Pitié-Salpêtrière Hospital, Sorbonne University, Paris, France; ^8^Neurodegenerative Disorder Research Center, Division of Life Sciences and Medicine, and Department of Neurology, Institute on Aging and Brain Disorders, University of Science and Technology of China and First Affiliated Hospital of USTC, Hefei, China; ^9^Department of Neurodegenerative Disease, UCL Institute of Neurology, London, United Kingdom; ^10^UK Dementia Research Institute at UCL, London, United Kingdom; ^11^Hong Kong Center for Neurodegenerative Diseases, Clear Water Bay, Kowloon, Hong Kong SAR, China; ^12^Wisconsin Alzheimer’s Disease Research Center, University of Wisconsin School of Medicine and Public Health, University of Wisconsin–Madison, Madison, WI, United States; ^13^Department of Biology and Microbiology, Rīga Stradinš University, Rīga, Latvia; ^14^Institute of Oncology and Molecular Genetics, Rīga Stradinš University, Rīga, Latvia; ^15^Outpatient Department, Pauls Stradiņš Clinical University Hospital, Rīga, Latvia; ^16^Department of Neurology, Pauls Stradiņš Clinical University Hospital, Rīga, Latvia

**Keywords:** systemic sclerosis, scleroderma, polyneuropathy, nervous system, autoimmune, serum biomarkers

## Abstract

**Introduction:**

Systemic sclerosis (SSc) is a rare autoimmune disease with multiple organ involvement; however, the contribution of the nervous system (NS) remains relatively understudied. There are no specific data on the role of the autoimmune response and inflammation in the development of peripheral nerve system (PNS) damage in SSc and markers to assess this damage have yet to be identified.

**Objectives:**

The primary objective of this study was to define the autoimmune mechanisms that lead to neuropathy by identifying antibodies (Abs) that target certain component of the NS or are associated with SSc. The secondary objective was to identify markers of NS damage that correlate with the detection and progression of polyneuropathy (PNP).

**Methods:**

This study included patients diagnosed with SSc who met ACR/EULAR 2013 classification criteria at two leading Latvian hospitals between January 2016 and December 2021. Patients underwent a nerve conduction study (NCS). The SSc-associated Abs, Abs against myelin-associated glycoprotein (MAG) and anti-ganglioside Abs (GM1, GM2, GD1a, GD1b and GQ1b) were analysed. Potential serum PNS biomarkers—neurofilament light chain (NfL), glial fibrillary acidic protein (GFAP), fibroblast growth factor 21 (FGF21) and growth/differentiation factor 15 (GDF15)—were measured.

**Results:**

We recruited 103 Caucasian patients diagnosed with SSc. SSc-associated Abs did not differ significantly between patients with and without PNP (*p* > 0.05). Anti-MAG and anti-ganglioside Abs in patients with PNP did not present a significant increase above the reference range. NfL, GFAP and GDF15 were significantly elevated in the presence of PNP (*p* < 0.05), with a moderate to high effect size (*r* = 0.36–0.65). Our regression analysis revealed a strong association between the HAQ-DI score, older age, male gender and the risk of developing PNP.

**Conclusion:**

The development of PNP in patients with SSc is most likely due to ageing, natural progression and the sequelae of the disease. Several serum biomarkers—NfL, GFAP and GDF15—could be used as relevant diagnostic biomarkers for PNP in patients with SSc. Future studies are warranted to validate the diagnostic efficacy of these biomarkers and to unravel the complex interplay of factors leading to PNP in patients with SSc.

## 1 Introduction

Systemic sclerosis (SSc) is a rare autoimmune disease with known autoantibodies that help establish a diagnosis and affect the prognosis (1–3). Although multiple organ involvement is widely acknowledged and studied, the contribution of the nervous system (NS) remains relatively understudied (4–6). In most recent classification criteria, NS damage was not included in point assessment, again highlighting its undefined role in SSc (7). Although a few studies have been conducted to establish the prevalence and type of NS involvement in SSc, mostly focusing on peripheral nervous system (PNS), they differed widely in numbers, partly because the authors used different methods of assessing NS damage. Over time, NS involvement in SSc has become more frequent, especially in recent studies, with a range from 17 to 40% (5, 8–11).

While only a few studies have evaluated the prevalence of NS involvement in SSc, there is even less research regarding the true pathogenesis of neuropathy in this rare disease. Most symptoms in patients with SSc can be explained by microvascular damage, the autoimmune response and inflammation, and fibrosis with variable severity (12, 13). The first and to this day the most accepted cause for neuropathy development in SSc is ischaemic damage of the NS (8, 14). Thus, it would be logical to conclude that patients with severe Raynaud’s disease, pitting scars and ischaemic skin lesions should develop neuropathy, but the proportion of patients without nerve damage contradicts this view, suggesting that other mechanisms are involved in the pathogenesis of neuropathy in SSc (15, 16).

There are no specific data on the role of the autoimmune response and inflammation in the development of neuropathy in SSc. In many systemic connective tissue diseases, the idea of studying specific antibodies (Abs) against various nerve structures comes from research performed in immune-mediated polyneuropathies (PNP) like Guillain–Barré syndrome (17, 18). This approach is still understudied in SSc and could lead to new insights into neuropathy pathogenesis and a future change in treatment tactics.

Another understudied issue is biomarkers for the progression and severity of SSc. Several biomarkers are used to measure and monitor the severity of lung and skin damage in SSc; however, markers to assess PNS damage and its progression have yet to be identified (19, 20). Neurofilament light chain (NfL) has proved to be useful biomarker for PNP, given that it is related to metabolic and genetic disorders, but it has not been studied in SSc (21, 22). There are other known biomarkers that are mostly or partly secreted from Schwann cells that can associated with PNS damage due to various diseases, including growth/differentiation factor 15 (GDF15) studied in diabetic neuropathies and glial fibrillary acidic protein (GFAP) associated with inflammatory PNP (23, 24).

The primary objective of this study was to define the autoimmune mechanisms that lead to neuropathy by identifying Abs that target certain component of the NS or are associated with SSc. The secondary objective was to identify markers of NS damage that correlate with the detection and progression of PNP.

## 2 Materials and methods

### 2.1 Subjects

This study included patients diagnosed with SSc who met the American College of Rheumatology/European Alliance of Associations for Rheumatology (ACR/EULAR) 2013 classification criteria and who received a consultation by rheumatologists at two leading Latvian hospitals between January 2016 and December 2021 (7). Using the hospital databases, patients with diagnostic codes M34.0–M34.9 based on the International Classification of Diseases, 10th Revision (ICD-10) were selected. Patients with connective tissue diseases other than SSc and patients with localised scleroderma were excluded. The age at disease onset was defined as the time of onset of the first non-Raynaud’s SSc symptom. The skin condition was evaluated according to the modified Rodnan skin score (mRSS) by a rheumatologist (25).

This study was approved by the Rīga Stradinš University medical ethics committee (Institutional Review Board reference no 22-2/481/2021). All participants provided written informed consent.

### 2.2 Methods

The enrolled subjects underwent a uniform evaluation of the PNS. First, the patients underwent a nerve conduction study (NCS) by a certified neurophysiology expert. Motor and sensory conduction were evaluated according to the PNP examination protocol (26). Each patient underwent an NCS of the bilateral upper extremities (the motor and sensory components of the ulnar and median nerves) and the bilateral lower extremities (the motor component of the peroneal and tibial nerves and the sensory component of the sural nerve) to determine nerve conduction latency, amplitude, and velocity. The patients with abnormal NCS results—considering the normal values used in Latvian clinical practice—in more than one attribute for two separate nerves were diagnosed as having PNP. The patients were divided in two groups according to the NCS results. The first group included patients with PNP, while the second included patients without PNP.

The patients were also evaluated with the Health Assessment Questionnaire Disability Index (HAQ-DI). The use of personal assistance or assistive devices were acknowledged. The scores from each of the eight sections were added together and then divided by eight to obtain the functional disability index. In addition, blood was collected from each patient. After separating the serum, aliquots were stored at −80°C prior to analyses.

The SSc-associated Abs were analysed using a commercial line immunoblot assay (EUROLINE Systemic Sclerosis Profile, Euroimmun). The EUROLINE Systemic Sclerosis (Nucleoli) Profile (IgG) contains 13 recombinant antigens: DNA-topoisomerase I (Scl-70), centromere proteins A and B (CENP-A and CENP-B, respectively), RNA polymerase III (subunits RP11 and RP155), fibrillarin, NOR-90, Th/To, PM-Scl-100, PM-Scl-75, Ku, platelet-derived growth factor receptor (PDGFR) and Ro-52. The detection and interpretation were carried out electronically using the Euroimmun EUROLineScan programme. A signal intensity of 0–5 (negative) and 6–10 (borderline) was considered negative, while a signal intensity of ≥11 was considered positive.

Several nervous system–specific Abs—namely Abs against myelin-associated glycoprotein (MAG) and anti-ganglioside Abs (GM1, GM2, GD1a, GD1b and GQ1b)—were evaluated with GanglioCombi^®^ MAG enzyme-linked immunosorbent assay (ELISA) kits (Bühlmann Laboratories). A signal intensity of 0–29 (negative) and 30–49 (borderline) was considered negative, while a signal intensity of ≥50 was considered positive. These Abs were assessed in patients with PNP first. If the data suggested a significant change in these patients, then the other groups were evaluated.

Two potential serum PNS biomarkers—NfL and GFAP—were measured with a Single molecule array (Simoa) assay (Quanterix, Billerica, MA, United States). Fibroblast growth factor 21 (FGF21) and GDF15 were measured using commercially available ELISAs according to the manufacturer’s instructions (R&D Systems, Minneapolis, MN, United States). All measurements were performed in one round of experiments using one batch of reagents by board-certified laboratory technicians who were blinded to the clinical data. The intra-assay coefficients of variation, determined using internal control samples, were below 10%.

### 2.3 Data analysis

The data distribution was assessed with a normal Q–Q plot and the Shapiro–Wilk test. The Mann–Whitney U test was used to compare SSc-associated Abs, NfL, GFAP, GDP-15 and FGF21 between patients with and without PNP. Additionally, this test was used to compare NfL between the control group and patients with PNP. Differences in SSc-associated Abs between patients with and without PNP were assessed with the chi-square test of homogeneity or Fisher’s test.

A binomial logistic regression was conducted to determine factors (age, sex, SSc duration, mRSS and HAQ-DI) related to patients with and without PNP. Forward and backward stepwise regression methods were used to build the model. All possible models and interactions were calculated. The Akaike information criterion (AIC) was used to select the best model. Additionally, receiver operating characteristic (ROC) curve analysis, to determine the area under the curve (AUC), was conducted to evaluate the performance of the regression model as binary classifier. An AUC >0.7 was considered to indicate good performance in distinguishing between patients with and without PNP. The Youden index was used to identify the optimal cut-off point.

## 3 Results

We initially recruited 103 Caucasian patients diagnosed with SSc (18 men and 85 women). Table 1 summarises the sex-specific clinical and Ab characteristics in these patients.

**Table 1:** Sex-specific clinical and antibody characteristics in patients with systemic sclerosis.

		Men	Women	Total
Descriptive statistic	Number of patients	18	85	103
	Mean (standard deviation) age in years	60.06 (14.92)	61.66 (11.95)	61.38 (12.46)
	Mean (standard deviation) disease duration in years	8.95 (6.33)	15.14 (9.87)	14.06 (9.62)
Symptoms	Raynaud’s phenomenon, *n* (%)	16 (88.88%)	71 (83.52%)	87 (84.46%)
	Mean (standard deviation) Modified Rodnan skin score	10.36 (12.95)	10.67 (8.78)	10.63 (9.41)
SSc-associated antibodies	Classical antibodies* *n* (%)	8 (44.44%)	52 (65.82%)	60 (61.86%)
	Scl-70 n (%)	4 (22.22%)	18 (22.78%)	22 (22.68%)
	CENP-A and CENP-B *n* (%)	4 (22.22%)	31 (39.24%)	35 (36.08%)
	RP11 and RP155 n (%)	0	3 (3.80%)	3 (3.09%)
	Novel antibodies** *n* (%)	9 (50%)	35 (44.30%)	44 (45.36%)

*Scl-70 (topoisomerase I), CENP-A and CENP-B (centromere proteins A and B, respectively), RP11 and RP155 (RNA polymerase III). **Fibrillarin, NOR-90, Th/To, PM-Scl-100, PM-Scl-75, Ku, platelet-derived growth factor receptor (PDGFR) and Ro-52.

Among the 103 patients recruited for this study, three declined to undergo an NCS. Following the NCS, the remaining cohort of 100 patients with SSc was stratified into subgroups based on the presence or absence of PNP. We identified PNP in 43 patients, representing 43% of the cohort. Within this subset, 15 patients had sensory-motor demyelinating PNP, while 28 had sensory-motor axonal demyelinating PNP. Table 2 illustrates the distinctions in demographic, clinical, and neurophysiological characteristics between patients with SSc and with or without PNP.

**Table 2:** Demographic, clinical and neurophysiological characteristics and comparisons of patients with systemic sclerosis and with or without polyneuropathy (PNP).

Variable	SSc without PNP 57 (57%)	SSc with PNP 43 (43%)	*p*-value
**Sex, n (%)**			0.0
Male	5 (29.41%)	12 (70.59%)	
Female	52 (62.65%)	31 (37.35%)	
**Mean (standard deviation) age in years**	57.30 (12.24)	67.07 (10.47)	<0.001
**Mean (standard deviation) disease duration in years**	12.48 (8.68)	16.26 (10.51)	0.049
**Mean (standard deviation) modified Rodnan skin score**	8.05 (9.14)	7.36 (9.67)	0.715
**Raynaud’s phenomenon, *n* (%)**	51 (89.47%)	36 (83.7%)	0.860
**Mean (standard deviation) nerve conduction study results**			
*Nervus peroneus*			
Amplitude (mV)	3.32 (1.79)	2.10 (1.28)	<0.001
Velocity (m/s)	45.2 (11.1)	41.7 (3.43)	<0.001
*Nervus tibialis*			
Amplitude (mV)	8.38 (2.84)	4.90 (2.84)	< 0.001
Velocity (m/s)	46.5(2.58)	40.8 (3.20)	<0.001
*Nervus suralis*			
Amplitude (mV)	11.7 (6.54)	7.54 (4.73)	0.002
Velocity (m/s)	47.2 (12.2)	41.1 (1.75)	<0.001

We assessed SSc-associated Abs in 97 patients; they did not differ significantly between patients with and without PNP (*p* > 0.05). We assessed anti-MAG and anti-ganglioside Abs in 24 patients. All 24 patients had PNP based on the NCS results, but they did not present a significant increase in the Abs above the reference range.

We assessed potential PNS serum biomarkers—NfL, GFAP, GDF15 and FGF21—in 68 patients, 30 with PNP, 38 without PNP. Table 3 summarises the comparison of serum biomarkers concentration between patients with and without PNP. NfL, GFAP and GDF15 were significantly elevated in the presence of PNP (*p* < 0.05), with a moderate to high effect size (*r* = 0.36–0.65). We observed the most pronounced difference for NfL, with significantly lower levels in control subjects (median = 5.2, interquartile range [IQR] 4.3–7.4) compared with those with PNP (median = 15.3, IQR 11.8–25.0; U = 35.0, *p* < 0.001, *r* = 0.93).

**Table 3:** Comparison of biomarker levels in patients with systemic sclerosis (SSc) and with or without polyneuropathy (PNP).

Parameter	SSc without PNP 38 (55.88%)	SSc with PNP 30 (44.11%)	*p*-value	*r*
	Median (interquartile range)	Median (interquartile range)		
NfL, pg/mL	9.8 (6.0–13.1)	15.3 (11.8–25.0)	<0.001	0.62
GFAP, pg/mL	77.1 (43.9–99.0)	100.5 (67.8–159.8)	0.011	0.36
GDF15, pg/mL	964.5 (705–1,389)	1681.5 (1303–2049)	<0.001	0.65
FGF21, pg/mL	130.7 (65.3–372.5)	148.3 (99.5–287.5)	0.501	NA

FGF21 Fibroblast growth factor 21; GDF15, growth/differentiation factor 15; GFAP, glial fibrillary acidic protein; NfL, neurofilament light chain; NA, not applicable; r, effect size (Cohen’s r).

**Figure 1:**
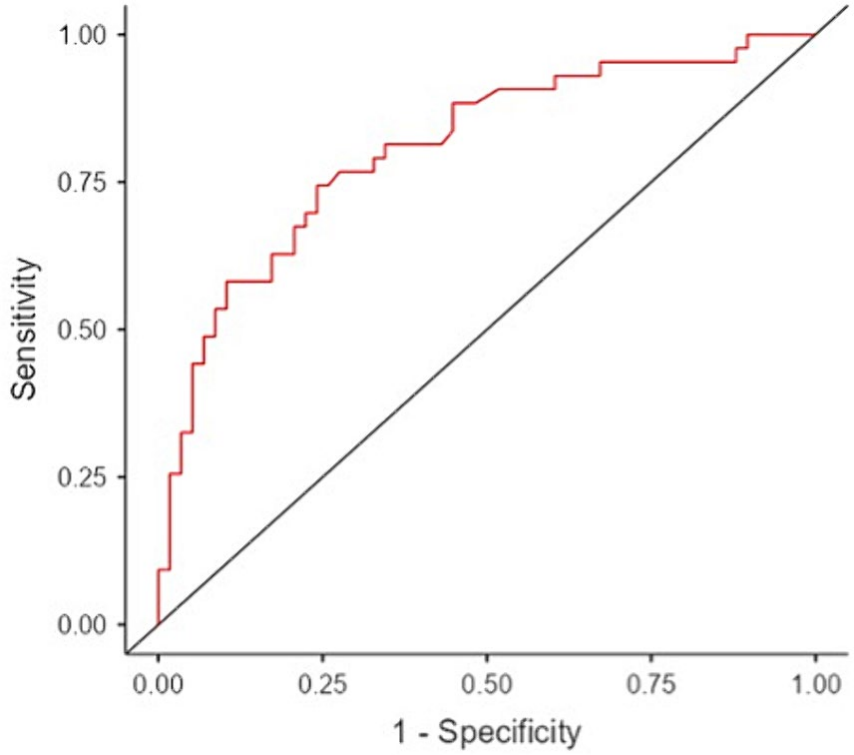
Performance of the final binomial logistic model based on receiver operating curve analysis that predicts polyneuropathy in patients with systemic sclerosis.

The final binomial logistic model was significant (χ^2^(3) = 30.8, *p* < 0.001; Table 4). The AUC was 0.81, indicating strong performance in distinguishing between patients with and without PNP (Figure 1).

**Table 4:** Results of final regression model showing the patient’s age, sex, and health assessment questionnaire disability index score as predictors of developing polyneuropathy.

					95% confidence interval of the odds ratio	
Predictor	Estimate	Z	*p*-value	Odds ratio	Lower	Upper
Intercept	−4.73	−3.11	0.002	0.01	0.001	0.17
Health assessment questionnaire disability index	0.67	2.39	0.017	1.95	1.13	3.36
Age	0.08	3.43	<0.001	1.09	1.04	1.14
Sex						
Female–Male	−2.00	−2.84	0.005	0.14	0.03	0.54

References: Dependent variable—patients without PNP; sex—male.

Our regression analysis revealed a strong association between the HAQ-DI score and the risk of developing PNP. A 1-point increase in the HAQ-DI score was significantly associated with a 95% higher likelihood of PNP (95% confidence interval [CI] 13–236%; *p* < 0.001). Based on the Youden index, individuals with an HAQ-DI score exceeding 0.63 had a greater than 50% probability of developing PNP. Age was also a significant predictor of PNP development. Each additional year of age was associated with a 9% increase in PNP risk (95% CI 4–14%; *p* < 0.001). Using the Youden index, individuals aged ≥63 years had a > 50% chance of developing PNP. Furthermore, we observed a significant sex difference in PNP risk. Women were 86% less likely to develop PNP compared with men (95% CI 46–97%; *p* < 0.001). Finally, we removed SSc duration and the mRSS from the final regression model due to their lack of statistical significance to the model.

## 4 Discussion

To our knowledge, this is one of the few studies on SSc that focuses on the involvement of the PNS, analysing both the prevalence of this complication and its pathogenesis and biomarkers of severity. We found a higher prevalence of PNP in SSc compared to data from other studies, possibly due to detailed and targeted assessment of the PNS. Moreover, the materials and methods used in these studies provide a wider range of results. A systematic review of 113 studies found a neuropathy prevalence of 27.37%, including 26% (*n* = 556/2143) with small fibre neuropathy and 10.8% (*n* = 231/2143) with large fibre neuropathy, however, titles and abstracts were not selected according to strict criteria for the neuropathies assessed (8). Confirmatory diagnostic tests for PNP in SSc varied according to study design (27–33). Some studies performed electrophysiological examinations, while others used imaging techniques, biopsies or other methods (4, 27–34). We believe that the high prevalence of PNP in our study can be explained by the fact that we worked with a relatively large study group and that all subjects were assessed using both clinical symptoms and electrophysiological methods, where motor and sensory components were studied on multiple nerves in each limb.

Historically, the classical SSc-specific or SSc-associated Abs—anti-topoisomerase Abs (ATAs), anti-centromere Abs (ACAs) and anti-RNA polymerase Abs (ARAs)—have received the most attention (35). Currently, novel Abs are assessed in addition to the classical Abs, and their presence in different clinical phenotypes remains a research goal (36). Only a few studies have evaluated the association of these classical Abs with neuropathies in SSc, and the results have varied greatly. In a 1994 study, 35% of patients with SSc presented neurological symptoms, and 73% of them had either ARAs or ATAs, but not ACAs (37). On the contrary, in a 2021 systemic review, the authors mentioned that ACAs are a risk factor for non-compression neuropathies in patients SSc (8). Similarly, in Brazilian study of 63 patients with SSc, seven were diagnosed with PNP, of whom 6 had ACAs and 1 had ARAs (38). In a Spanish study, ARAs, ATAs and ACAs were present in patients with SSc and PNP, but the authors did not provide the statistical analysis (39).

Expanded SSc-associated Ab panels have started to play an increasingly important role in research and clinical practice. Although there is wide spectrum of clinical phenotypes in SSc, information regarding NS involvement is frequently missing (40). We could not find published data about expanded SSc-associated Abs in patients with SSc and NS damage. Interestingly, none of our patients was positive for anti-PDGFR Abs, and only one patient was positive for anti-Fib Abs. The most common SSc-associated Abs were anti-Ro52 Abs, ACAs and ATA. Only three patients (3%) were positive for ARAs, a lower frequency than for Abs that are not included in the SSc classification criteria: anti-Ku, anti-PM100, anti-Th/To and anti-NOR90 Abs. We did not find significant association between any of the SSc-associated Abs and the presence of PNP.

In autoimmune neuropathies, gangliosides are one of the most frequent targets of Abs (41). Gangliosides are nerve fibre glycoproteins that play an important role in both impulse transmission and nerve fibre regeneration. Anti-ganglioside Abs are often detected in the serum of patients with Guillain–Barré syndrome (37–78% of the cases) (42). They have been studied in patients with systemic lupus erythematosus and neuropsychiatric manifestations: the authors detected Abs more frequently in patients with neuropsychiatric manifestations compared with the asymptomatic group (43). There are very few studies on anti-ganglioside Abs in patients with SSc. In 1994, 34 patients with scleroderma, of whom 28 had PNP, were evaluated for the presence of anti-GM1 Abs. The levels were lower in these patients compared with healthy individuals, and there was no association with the development PNP (44). In our study, performed almost 30 years later, we also could not find a significant association between anti-MAG or anti-ganglioside Abs and the development of PNP in patients with SSc. Due to the lack of data on the association between PNP in SSc and specific nervous system–specific Abs we initially determined Abs only in a subset of patients with definite PNP, randomly selected. We would most likely not expect a significant change if Abs were detected in all patients with PNP, and even if they were detected at low titres, these data would only show false positives and unnecessarily confound the overall significance of the study.

In this study, no Abs were associated with a more frequent development of PNP in patients with SSc. At present, immune-mediated peripheral nerve damage in SSc remains questionable. In the treatment of PNP in patients with SSc, the role of immunosuppressive drugs remains equivocal and, according to our data, there is no reason to expect them to be efficacious. Additional research is necessary to predict PNS damage in patients with SSc so that they can be managed appropriately.

In recent years, successful new candidate serum biomarkers have been identified for SSc-associated interstitial lung disease (ILD), including surfactant protein D (SP-D), Krebs von den Lungen 6 glycoprotein (KL-6), CCL18 and intercellular adhesion molecule 1 (ICAM-1) (45, 46). For ILD, there has been a focus on searching for biomarkers in SSc that are also related to skin involvement and vascular injury (20, 47). Unfortunately, researchers have not yet evaluated serum biomarkers for PNS damage in patients with SSc. Thus, we chose to evaluate the most promising biomarkers based on the connection to the PNS. Of these four serum biomarkers—NfL, GFAP, GDF15 and FGF21—three of them showed promise as candidate PNP serum biomarkers in patients with SSc.

NfL stand out as novel biomarker for early diabetic sensorimotor PNP; there are possible similarities in vascular injury in both diabetic PNP and PNP in SSc (21). Our findings confirmed the already established significant role of NfL as a serum biomarker for neuropathies of different aetiologies (48).

A less-studied biomarker in PNP is GFAP, which has mostly been associated with central NS damage due to its predominant secretion from astrocytes. However, studies have demonstrated the presence of GFAP in the PNS (49, 50). Researchers have reported elevated serum GFAP levels in chronic neuropathies like chronic sensory-motor axonal neuropathy and chronic inflammatory demyelinating PNP (24). Unlike NfL, GFAP has not been widely evaluated in diabetic neuropathies, reducing the likelihood of linking this biomarker to neuropathy caused by vascular injury. We did not find any studies of GFAP in SSc, but serum GFAP was significantly elevated in patients with SSc and PNP.

GDF15 and FGF21 have less association with the NS. GDF15 is a cytokine belonging to the transforming growth factor beta superfamily. Elevated GDF15 levels are observed in inflammation, myocardial ischaemia and tumours (51). Serum GDF15 levels were elevated in patients with pulmonary hypertension (PH) and SSc compared with patients with SSc but not PH, as well as in patients with SSc, ILD and more pronounced skin lesions (52–54). There is evidence of increased GDF15 secretion by Schwann cells in nerve injury, and increased GDF15 levels have been found in patients with diabetic neuropathy, mainly with more pronounced manifestations of metabolic syndrome (23, 55, 56). We found elevated serum GDF15 levels in the patients with SSc and PNP compared with the patients with SSc but not PNP. Of note, there have been no other studies that evaluated this serum biomarker in patients with SSc and neuropathies.

Only FGF21 showed no significant change between the SSc with PNP and the SSc without PNP groups. This pleiotropic hormone—considered to be a major regulator of energy homeostasis—is mainly synthesised in the liver, pancreas and adipose tissue (57, 58). Recently, researchers have shown that FGF21 has regenerative capability in the PNS by suppressing oxidative stress, and the FGF21 levels were elevated in patients with diabetic neuropathy after aerobic training (59, 60). While there have been no studies on FGF21 levels in patients with SSc, we found that FGF21 levels did not change significantly in patients with SSc and PNP, indicating that FGF21 has less of a connection to the NS compared with other biomarkers. FGF21 expression is significantly increased in the muscles of mice with mitochondrial myopathies, where its levels are directly related to the presence of cytochrome oxidase negative fibres, a marker associated with the severity of the disease. This observation underscores the relevance of FGF21 in muscle pathology, especially under conditions characterised by damaged mitochondrial function (61, 62).

We found that the axonal demyelinating form of PNP was the most common in our patients with SSc. The absence of significant correlations between Abs and PNP has led us to consider alternative pathogenic mechanisms. Comparisons between the patients with and without PNP showed several intriguing differences: the patients with PNP were generally older, with an average age of 67 years compared with 57 years, and it was more prevalent in men (66% compared with 36%). These observations indicate that ageing, metabolic factors and ischaemic mechanisms may contribute significantly to the emergence of axon neuropathies, reflecting the patterns observed in cases of idiopathic PNP. In the literature, researchers have noted a higher prevalence of idiopathic PNP in people aged >60 years. Similar results have been reported in studies focusing on chronic axon idiopathic PNP in people aged >60 years, with a 3:2 male-to-female ratio (63, 64). As the name suggests, the condition is idiopathic, and metabolic factors are most strongly considered to be involved in the aetiology, but microvasculopathy identified in biopsies shows a different pattern than in diabetic neuropathies (64, 65). These coincidences lead us to suspect sequential development of PNP in patients with SSc over time, associated with ageing and a logical progression of the disease with more pronounced vasculopathy and metabolic factor–associated effects. Our regression analysis confirmed this view: it showed that age is a significant predictor of PNP development.

A deeper look into the serum biomarkers we evaluated in patients with SSc revealed three biomarkers associated with PNP. NfL and GFAP had already been shown to be associated with axonal injury, strengthening our above hypothesis of the development of PNP in SSc (24, 66). On the other hand, GDF15 and FGF21 have mostly been associated with mitochondrial stress and subsequent metabolic changes (67, 68). Interestingly, they behaved differently in our study. While the FGF21 levels were slightly higher in patients with SSc and PNP, the difference was not significant. The GDF15 levels were significantly elevated in patients with SSc and PNP, similarly to patients with diabetic neuropathies, were metabolic damage plays an important role (23). We believe additional studies that detect muscle damage and loss are needed to further investigate the role of mitochondrial damage and metabolic markers in patients with SSc.

Our results suggest that the use of serum biomarkers in clinical environments may facilitate early identification of PNS damage in patients with SSc. By dynamically monitoring biomarkers such as the NfL, GFAP and GDF15, it could be possible to detect deterioration of nerve function without further electrophysiological testing. However, research focusing on hereditary neuropathy has challenged the effectiveness of neurofilament fluctuations as indicators of disease progression, suggesting that these markers may not be suitable for tracking slow-moving diseases due to their lack of specificity and their tendency to reflect general rather than specific nerve damage (69).

A strength of this study is the choice of the group of interest: PNP is one of the complications of SSc that seems to have been neglected. To our knowledge, this is the first study that has extensively defined serum tests of different significance in patients with SSc and PNP. Moreover, we analysed both the immune pathogenesis of PNP and the reflection of nervous system damage in serum biomarkers in a univariate manner. However, several limitations must be acknowledged. First, the study did not include a healthy control group, which might have provided more evidence for our findings linking the development of PNP in SSc patients also to natural ageing. Secondly, this study focused on the development of neuropathy as the main complication of SSc, without providing a full description of the patients’ other organ involvement such as ILD, PH and others. We included the presence of Raynaud’s phenomenon, which partially characterises vasculopathy, and the mRSS, which partially characterises disease severity by skin involvement, but it would also be very useful to include more clinical symptoms. However, the relationship of the different clinical manifestations of the disease to the involvement of the PNS must be demonstrated in future projects.

## 5 Conclusion

There was no association between SSc-associated or other inflammatory neuropathy-associated Abs and the development of PNP in patients with SSc. The development of PNP in patients with SSc is most likely due to ageing, natural progression and the sequelae of the disease. Several serum biomarkers—NfL, GFAP and GDF15—could be used as relevant diagnostic biomarkers for PNP in patients with SSc. Future studies are warranted to validate the diagnostic efficacy of these biomarkers and to unravel the complex interplay of factors leading to PNP in patients with SSc. This endeavour should ultimately pave the way for novel therapeutic strategies and a more nuanced understanding of this multifaceted disease.

## Data availability statement

The raw data supporting the conclusions of this article will be made available by the authors, without undue reservation.

## Ethics statement

The studies involving humans were approved by the Rīga Stradinš University medical ethics committee (Institutional Review Board reference no 22-2/481/2021). All participants provided written informed consent. The studies were conducted in accordance with the local legislation and institutional requirements. The participants provided their written informed consent to participate in this study.

## Author contributions

KI: Writing – original draft, Writing – review & editing. MZ: Writing – review & editing. KB: Writing – review & editing. HZ: Writing – review & editing. NK: Writing – original draft, Writing – review & editing. VĶ: Writing – original draft, Writing – review & editing.
